# Development and Analysis of a Sustainable Interlayer Hybrid Unidirectional Laminate Reinforced with Glass and Flax Fibres

**DOI:** 10.3390/polym17141953

**Published:** 2025-07-16

**Authors:** York Schwieger, Usama Qayyum, Giovanni Pietro Terrasi

**Affiliations:** Mechanical Systems Engineering Laboratory, Swiss Federal Laboratories for Materials Science and Technology, Überlandstrasse 129, CH-8600 Dübendorf, Switzerland; usama.qayyum@empa.ch (U.Q.); giovanni.terrasi@empa.ch (G.P.T.)

**Keywords:** natural fibres, hybrid unidirectional FRP composites, interlayer hybrids, pseudo-ductility, fibre optic strain sensing, distributed fibre optic sensing (DFOS)

## Abstract

In this study, a new fibre combination for an interlayer hybrid fibre-reinforced polymer laminate was investigated to achieve pseudo-ductile behaviour in tensile tests. The chosen high-strain fibre for this purpose was S-Glass, and the low-strain fibre was flax. These materials were chosen because of their relatively low environmental impact compared to carbon/carbon and carbon/glass hybrids. An analytical model was used to find an ideal combination of the two materials. With that model, the expected stress–strain relation could also be predicted analytically. The modelling was based on preliminary tensile tests of the two basic components investigated in this research: unidirectional laminates reinforced with either flax fibres or S-Glass fibres. Hybrid specimens were then designed, produced in a heat-assisted pressing process, and subjected to tensile tests. The strain measurement was performed using distributed fibre optic sensing. Ultimately, it was possible to obtain repeatable pseudo-ductile stress–strain behaviour with the chosen hybrid when the specimens were subjected to quasi-static uniaxial tension in the direction of the fibres. The intended damage-mode, consisting of a controlled delamination at the flax-fibre/glass-fibre interface after the flax fibres failed, followed by a load transfer to the glass fibre layers, was successfully achieved. The pseudo-ductile strain averaged 0.52% with a standard deviation of 0.09%, and the average load reserve after delamination was 145.5 MPa with a standard deviation of 48.5 MPa. The integrated fibre optic sensors allowed us to monitor and verify the damage process with increasing strain and load. Finally, the analytical model was compared to the measurements and was partially modified by neglecting the Weibull strength distribution of the high-strain material.

## 1. Introduction

Fibre-reinforced polymers (FRPs) offer many characteristics that are useful in different fields of engineering. They can be designed to have a high tensile strength while still being lightweight. FRPs are also highly resistant to corrosion compared to many metals [1]. A disadvantage of FRPs is their brittle failure behaviour. This means that there are no apparent warning signs that an FRP is close to failure. Unidirectional (UD) FRPs in particular do not show any appreciable damage before complete failure. This is the key problem that unidirectional hybrid materials aim to solve. By combining different fibre materials within a laminate, it is possible to achieve more gradual failure behaviour, avoiding the usual abrupt and brittle failure of UD FRPs.

**Figure 1:**
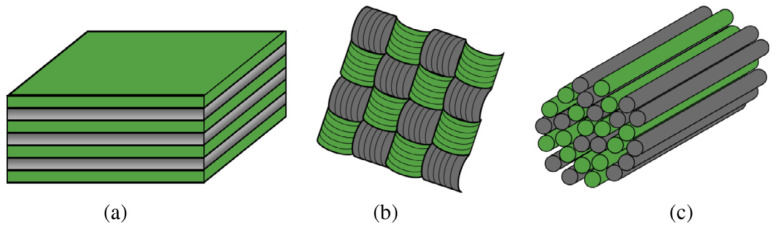
Three types of hybridisation: (**a**) Interlayer. (**b**) Intralayer. (**c**) Intrayarn. Reproduced with permission from Yentl Swolfs, Compos. A: Appl. Sci. Manuf., Elsevier, 2014 [2].

There are three main methods through which hybridisation can be realised within a fibre-reinforced polymer composite [2]. An interlayer hybrid consists of multiple layers of reinforcement plies, where each ply contains only one type of fibre; see Figure 1a. For an intralayer hybrid, at least two types of fibres must be interwoven within one ply of material; see Figure 1b. An intrayarn hybrid contains twines of fibres, where multiple types of fibre are spun into a single yarn; see Figure 1c.

Through interlayer hybridisation of a composite laminate, it is possible to achieve pseudo-ductile behaviour in tensile tests. This is achieved by combining two types of fibre, where one has a lower breaking strain than the other. During the tensile test, the low-strain (LS) material breaks first, while the high-strain (HS) material is able to carry the load after the initial failure of the LS material.

**Figure 2:**
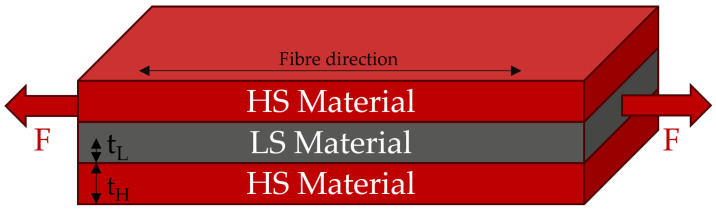
General form of the unidirectional FRP hybrids used in this study. $t_{H}$ and $t_{L}$ are the half-thicknesses of the HS and LS materials, respectively.

In the last decade, failure behaviour has been investigated thoroughly for unidirectional interlayer hybrids in several research projects, which have mainly investigated glass/carbon [3,4,5,6,7,8] and carbon/carbon [9,10,11] hybrids. Those studies all used a specific type of interlayer hybrid, composed of one layer of LS material in the centre sandwiched between layers of HS material on the top and bottom. The general form of such a hybrid can be seen in Figure 2.

**Figure 3:**
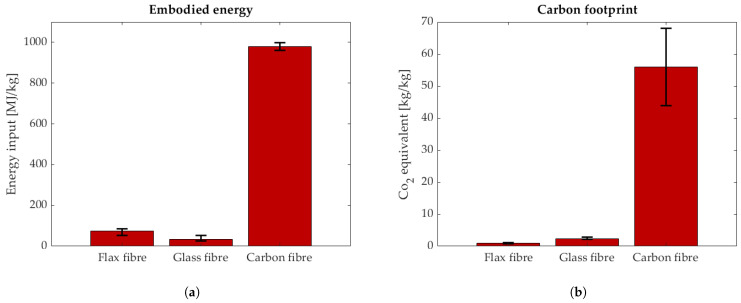
Environmental impact of different types of fibres [14,15,16,17]. (**a**) Embodied energy per kg of dry fibre. (**b**) CO_2_ emissions in kg per kg of dry fibre.

The problem of carbon/carbon and carbon/glass hybrids is the high environmental impact of the fibres. The embodied energy and the CO_2_ emissions associated with production are significantly higher for carbon fibres than for flax and glass fibres. This is displayed in Figure 3. Glass and flax fibres have a similar carbon footprint; see Figure 3. In construction, polymers reinforced with pure glass fibre are extensively used as concrete reinforcement due to their non-magnetic properties, durability, and light weight [12]. In that application, the safety factors for tensile loads are very high. Depending on the safety standard, the sustained service stress must be three to four times lower than the tensile stress [13]. This is due to stress corrosion (alkaline embrittlement under sustained tensile stress) and the brittle failure behaviour of glass FRPs. This could be a potential use case where a pseudo-ductile material could be used with lower safety factors due to its gradual failure behaviour.

**Figure 4:**
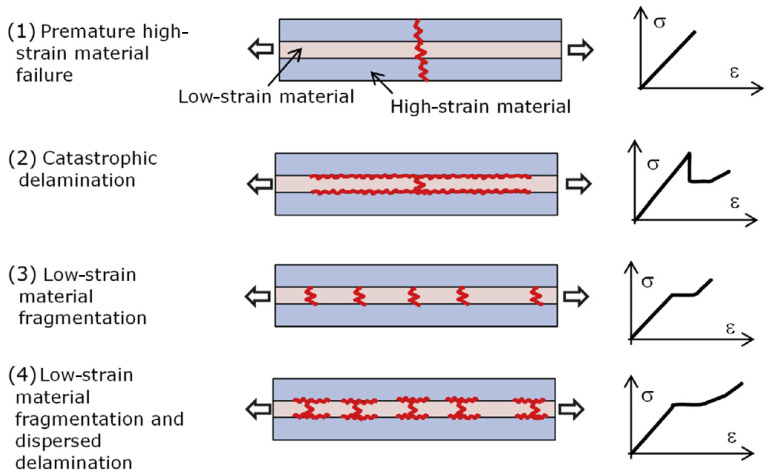
Four different damage-modes for UD interlayer hybrids [5].

To understand the failure mechanisms of interlayer hybrid materials and to predict the failure sequence, an analytical model was proposed by Jalalvand et al. [3,5]. The damage-modes are presented as four different scenarios: premature failure, catastrophic delamination, LS material fragmentation, and LS material fragmentation with dispersed delamination. The different damage-modes are summarised in Figure 4.

**Figure 5:**
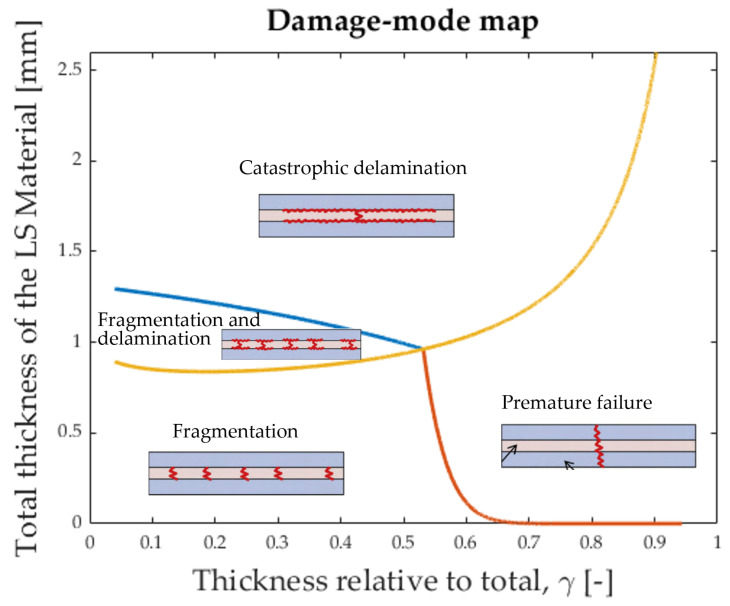
Damage mode map based on [3].

To achieve pseudo-ductility in tension, it is fundamental to reliably predict the damage-mode for a chosen hybrid laminate. For this reason, the model by Jalalvand et al. [3] proposes a damage-mode map that can be constructed for a specific combination of fibres. The damage-mode map then indicates what damage-mode is to be expected for a certain combination of layer thicknesses, see Figure 5.

The damage-mode for which the hybrids were designed in this study is ’catastrophic delamination’. This damage-mode represents an efficient way to obtain a warning from the laminate when overloaded, since the initial failure of the LS material is accompanied by a drop in stress and followed by constant stress during delamination. This is the warning sign that is wanted before complete failure of the specimens, which should ideally occur after a further load increase (called load reserve).

In this study, a flax/epoxy laminate was used as the LS material, and glass/epoxy outer layers were used as the HS material. This material combination has never been investigated before with the goal of achieving pseudo-ductile failure behaviour. Other studies investigated flax/carbon hybrid behaviour [18] to improve the strength of flax composites. Additionally, a study on flax/glass hybrids under tension was been carried out by Zhang et al. [19]. There, the stacking sequence of glass and flax layers was different from the sequence used in this study, and the goal was to improve the tensile strength of the laminate by altering the stacking sequence without aiming to achieve pseudo-ductile stress–strain behaviour in tension.

## 2. Materials and Methods

In order to construct the damage-mode map for the glass and flax fibres used herein, their mechanical properties need to be known. Through preliminary tests at EMPA, the properties in Table A1 were found.

**Figure 6:**
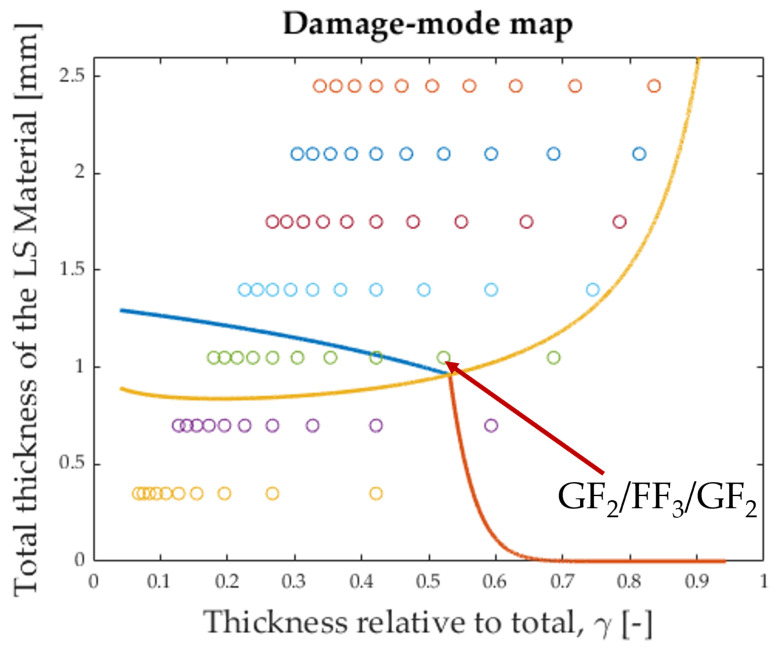
Damage-mode map for glass/flax hybrids. Every circle corresponds to a possible hybrid configuration based on the layer thicknesses given in Table A1. The chosen configuration for this study is marked with an arrow. The colour of the circles corresponds to the number of LS-material plies. (From the bottom up: 1 (purple) to 7 (yellow)).

Based on these properties and considering the material and geometric parameters given in the literature and summarised in Table A2, it was possible to construct a damage-mode map, shown in Figure 6. The boundary lines between the different damage-modes were constructed using the formulas proposed by [3]. (α is the ratio of Young’s moduli, β is the ratio of thicknesses, and γ is the LS material thickness fraction. For more information, see Section Abbreviations).

The blue line in Figure 6 represents the following formula, rearranged from [3]:

(1) $$t_{L}=\frac{2G_{IIC}E_{H}}{S_{L}^{2}}\frac{\alpha \left(1-\gamma \right)}{\alpha \gamma +1-\gamma }$$

The orange line in Figure 6 represents the following formula, rearranged from [3]:

(2) $$\sqrt[{m}]{t_{L}}=\frac{S_{H}}{K_{t}S_{L}}\left(\frac{\alpha }{\alpha \beta +1}\right)\sqrt[{m}]{\frac{\beta }{2WL}}$$

The yellow line in Figure 6 represents the following formula, rearranged from [3]:

(3) $$t_{L}^{\left(1/2-1/m\right)}=\beta^{\left(-1/m\right)}\frac{K_{t}}{S_{H}}\sqrt[{m}]{2LW}\sqrt{2G_{IIC}E_{H}\frac{1+\alpha \beta }{\alpha }}$$

The configuration $\left[GF_{2}/FF_{3}/GF_{2}\right]$ was chosen because its proximity to the intersection of the boundary lines leads to relatively high pseudo-ductile strain [3].

The analytical stress–strain curve for $\left[GF_{2}/FF_{3}/GF_{2}\right]$ was constructed using the model proposed by Jalalvand et al. [5]. For this model, the five points indicated in Table 1 needed to be evaluated.

To calculate the points in Table 1, the following formulas were proposed by Jalalvand et al. [5].

**Figure 7:**
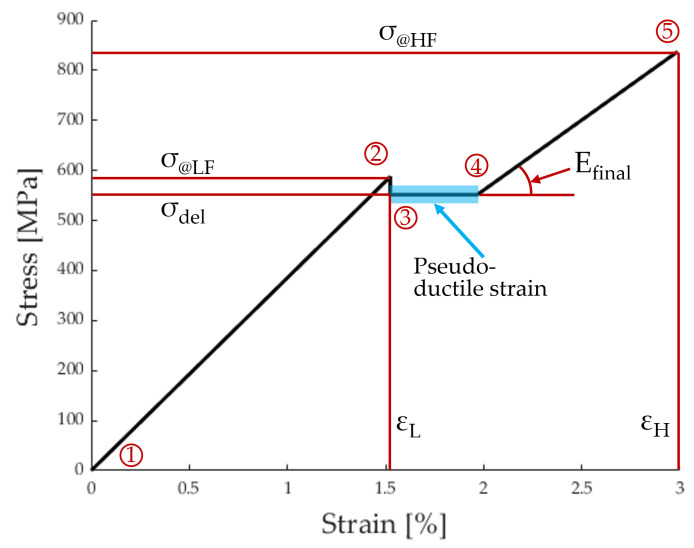
Plot of the analytic model with the 5 points from Table 1.

The laminate stress at which the LS material fails, marked by Point 2 in Figure 7, is as follows:

(4) $$\sigma_{@LF}=S_{L}\frac{\alpha \beta +1}{\alpha (\beta +1)}$$

The stress level at which the LS material delaminates from the HS material, marked by Points 3 and 4 in Figure 7, is as follows:

(5) $$\sigma_{del}=\frac{1}{1+\beta }\sqrt{\frac{1+\alpha \beta }{\alpha \beta }\frac{2G_{IIC}E_{H}}{t_{H}}}$$

The HS material finally fails at the stress marked by Point 5 in Figure 7. Its value can be expressed as follows:

(6) $$\sigma_{@HF}=\frac{S_{H}}{K_{t}\sqrt[{m}]{V}\left(1+\beta \right)}$$

The Young’s modulus of the laminate after complete delamination [5] is as follows:

(7) $$E_{final}=\frac{E_{h}}{1+\beta }$$

**Figure 8:**
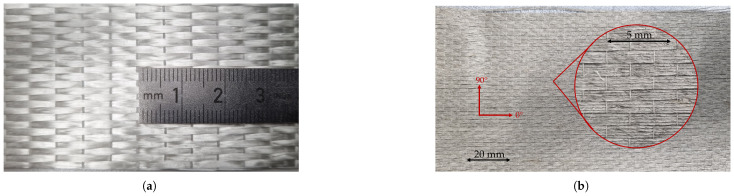
Dry fabrics used for manufacturing the hybrids: (**a**) Beihai S-Glass UD fabric, 414 gsm [20]. (**b**) Swiss Composites flax UD fabric, 300 gsm [21].

The hybrid laminate was manufactured using UD fabrics for both the glass and flax fibres; see Figure 8.

The fabric was cut to size and impregnated by hand using Araldite LY 5052/Aradur 5052 (Huntsman, Basel, Switzerland). The wet laminate was then placed into a mould with the exact dimensions intended for the final specimen: 24 mm in width and 280 mm in length. To cure the epoxy, the mould was placed in a heating press at 80 °C and subjected to 15 bar of pressure. After 20 min, the specimen was ready to be taken out of the mould. All the specimens were post-cured for at least 4 h at 100 °C in an oven without additional pressure.

**Figure 9:**
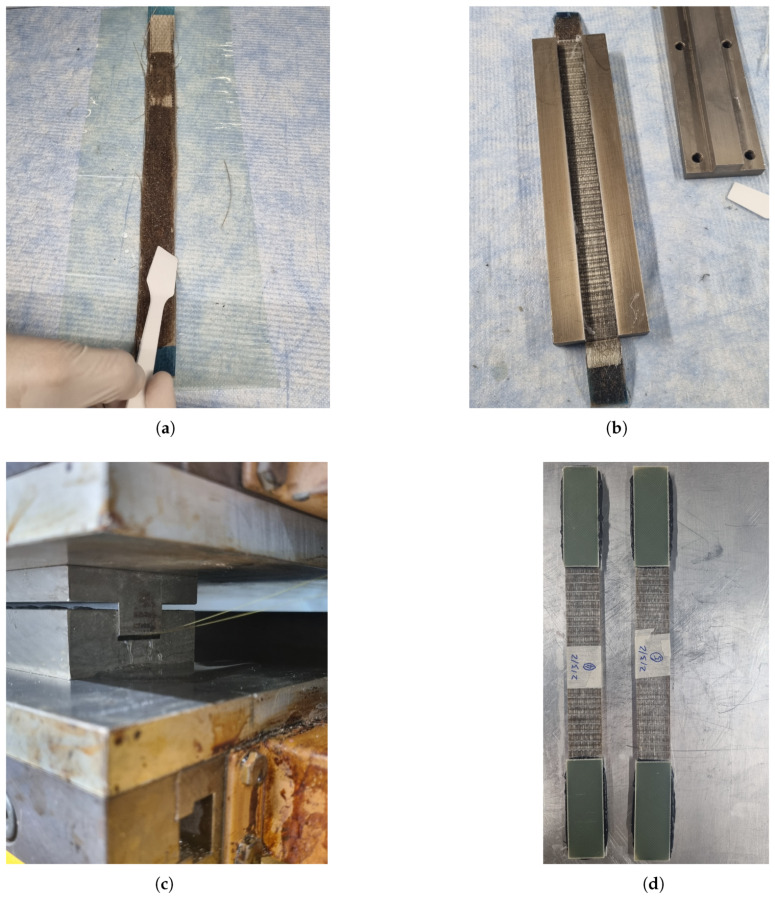
Manufacturing process for the specimens: (**a**) Hand lamination of a specimen. (**b**) Mould with the specimen inside; the lid of the mould can be seen on the right. (**c**) Mould inside the hot press for the initial curing cycle at 80 °C and 15 bar. (**d**) Finished specimens with end tabs installed.

End tabs made of ±45° GFRP with a thickness of 2 mm and a length of 70 mm were glued to all the specimens with 3M DP490 epoxy glue (3M, Saint Paul, MN, USA) This way, the hydraulic grips of the testing machine were not in direct contact with the specimen, and the end tabs ensured a good surface to grip. The process can be seen step by step in Figure 9.

**Figure 10:**
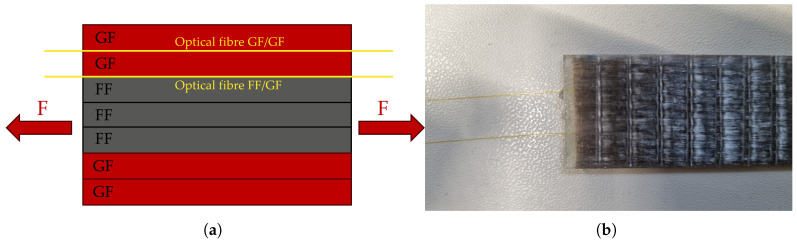
Visualisation of the optical fibres. (**a**) A diagram of the interlayer hybrid laminate $\left[GF_{2}/FF_{3}/GF_{2}\right]$, showing the locations of the optical fibres within the laminate. (**b**) A finished specimen with optical fibres (yellow).

Within the specimens, optical fibres were embedded to measure the strain accurately during the tensile tests. This method has been applied successfully by Martinoni et al. to measure the strain distribution in all-carbon hybrids [10]. The positions of the optical fibres in the hybrid laminates are shown in Figure 10. The measuring method used was Rayleigh backscattering [22,23], implemented with the ODiSi 6104 interrogator (Luna Innovations Inc., Roanoke, VA, USA) This method allows high-spatial-resolution measurement of the strain distribution along the fibre, with intervals of 0.65 mm between measurements.

The tensile tests were carried out using a Z250 (ZwickRoell, Ulm, Germany) universal testing machine. and followed the EN ISO 527-5 testing standard [24]. The specimens were clamped with hydraulic grips, which ensured that the specimens did not slip during testing. The applied pressure was 120 bar. The dimensions of the specimens were slightly modified compared to the EN ISO 527-5 specifications. The dimensions used in this study resulted from the geometry of the available mould and the specific layer thicknesses that needed to be used to achieve the desired damage-mode. The dimensions used for the tensile tests are summarised in Table 2. All the samples produced for testing and microscopy are listed in Table 3. EN ISO 527-5 requires a minimum of 5 samples for the tensile tests; one more was produced because the thickness of specimen 4 differed greatly from the others for unknown reasons. A 7th sample was produced to investigate the internal structure of the hybrid laminate under a microscope.

## 3. Results

### 3.1. Average Strain Along the Specimens

**Figure 11:**
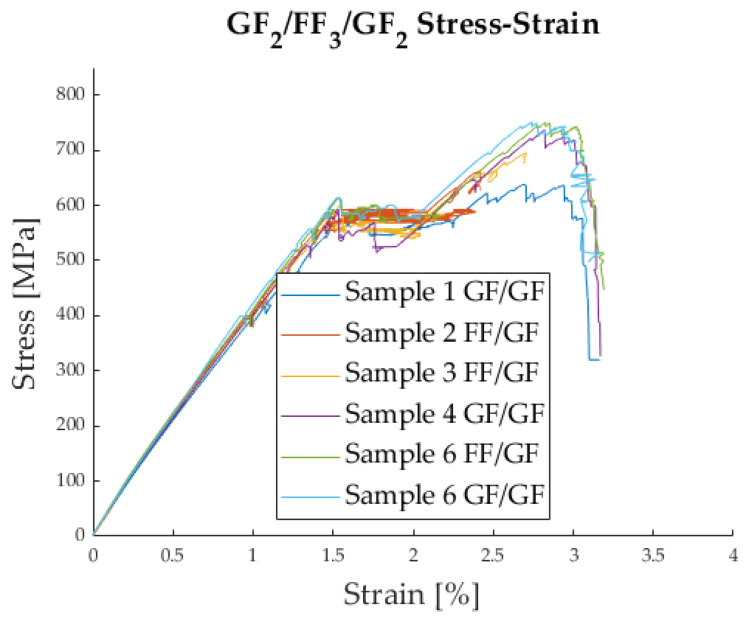
Average strain along the specimen, measured with DFOS.

To obtain the typical stress–strain plot for the tensile tests, the average strain along the length of the specimen can be measured by computing the mathematical average of the DFOS strain values captured every 0.65 mm along the free length of the specimens (140 mm). This can be seen in Figure 11.

**Figure 12:**
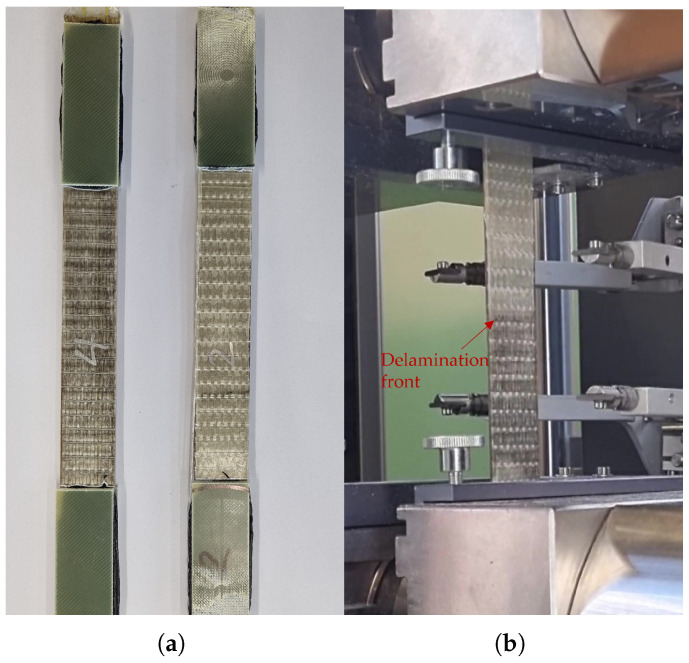

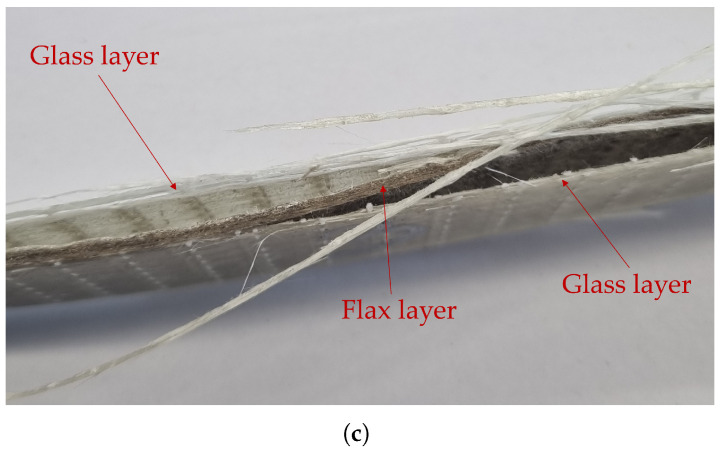
Images from the delamination process. (**a**) Specimen 2 (on the right) was delaminated after the tensile test. Specimen 4 (on the left) had not been tested. (**b**) A specimen during the tensile test with a visible delamination front, where the colour changes in the middle. (**c**) Side view of a delaminated specimen with intact outer Glass layers.

It was possible to observe the delamination process visually during the tensile tests; see Video S1 of the Supplementary Material. The delamination changes how the light is refracted within the sample, leading to a change in the colour of the specimen. This can be seen in Figure 12a,b. For Figure 12a,c, the specimen was taken out of the testing machine shortly before final failure of the outer glass fibre layers. The average stress and strain levels that were obtained are listed in Table 4.

### 3.2. Strain Distribution

**Figure 13:**
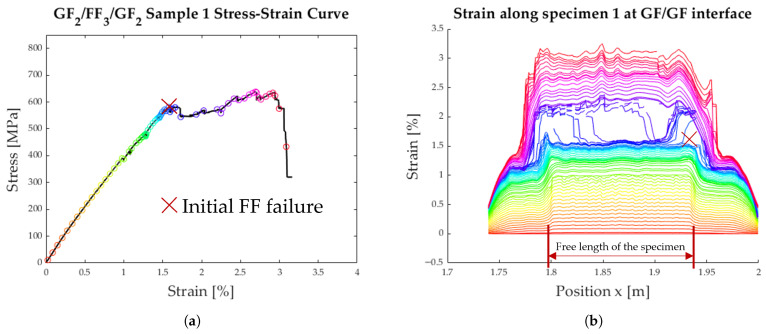
Results from tensile testing of specimen 1 with DFOS; the initial FF layer failure is marked with a red X. (**a**) Average strain of the specimen. (**b**) Strain distribution along the specimen.

**Figure 14:**
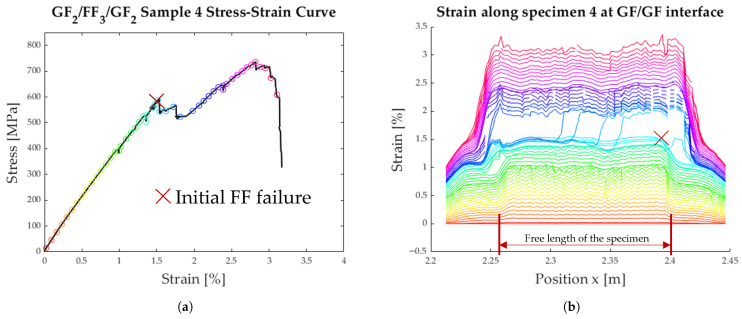
Results from tensile testing of specimen 4 with DFOS; the initial FF layer failure is marked with a red X. (**a**) Average strain of the specimen. (**b**) Strain distribution along the specimen.

**Figure 15:**
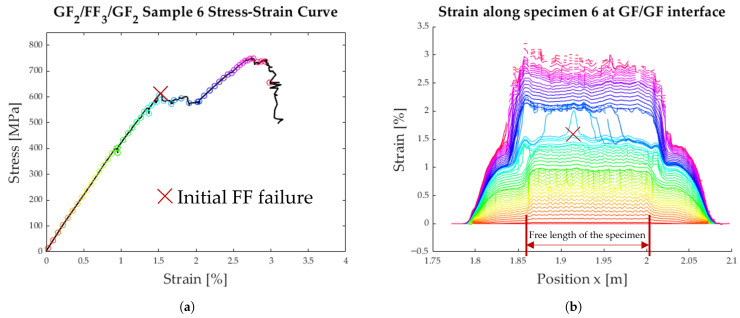
Results from tensile testing of specimen 6 with DFOS; the initial FF layer failure is marked with a red X. (**a**) Average strain of the specimen. (**b**) Strain distribution along the specimen.

The strain along the length of the specimen can be seen in Figure 13b, Figure 14b, and Figure 15b. Each coloured line represents one instant in time when the strain was measured. The measured strain distribution was smoothed to fill small gaps in the data, using a moving average over seven data points and discarding the empty entries. These time steps are also synchronised with the measured stress–strain curves in Figure 13a, Figure 14a, and Figure 15a.

### 3.3. Microscopy

**Figure 16:**
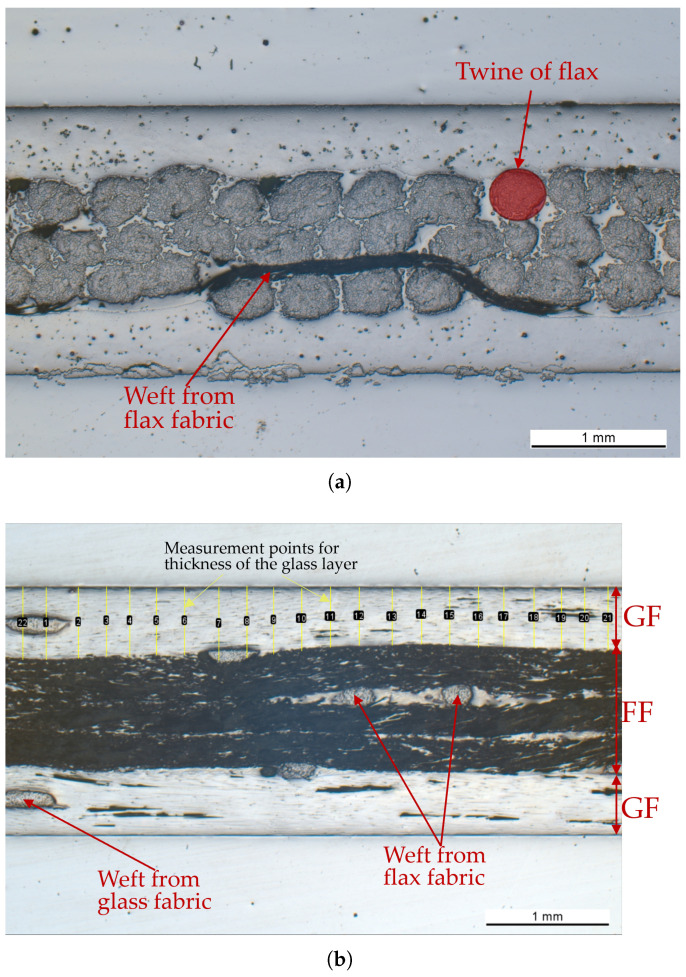
Microscope images: (**a**) Transverse cut of the specimen, showing the cross-sections of the individual fibres and fibre bundles (twines). (**b**) Longitudinal cut of the specimen, showing a cross-section of the weft.

Images from the optical microscopy analysis can be seen in Figure 16. The images, which were taken using a Zeiss Axio Imager 2(Carl Zeiss AG, Oberkochen, Germany), were used to investigate the internal structure of the hybrids and evaluate the manufacturing quality. It was also possible to more accurately measure the individual layer thicknesses of the glass and flax plies; see Table 5. Images from longitudinal and transverse sections were used to measure the thickness of each layer.

### 3.4. Additional Testing of the Individual Materials

The preliminary tests for the characterisation of the HS and LS materials were performed using a slightly different specimen manufacturing process than used for the hybrids. For the preliminary tests, a vacuum-assisted resin transfer method was used to manufacture a sheet of laminate. From this sheet, the specimens were then cut according to the EN ISO 527-5 standard. The mechanical properties obtained from the preliminary UD layer characterisation are shown in Table A1. The hybrid laminates that were tested in this study were manufactured using the hot press procedure described above to achieve a higher fibre volume fraction. To determine the mechanical properties of the glass and flax laminates more accurately, tensile tests were carried out with pure glass and pure flax laminates, using the same manufacturing process as described for the hybrids. The results of those tests are summarised in Table 6. Since the manufacturing process can influence the fibre alignment and the fibre volume fraction, the mechanical properties of the two laminates changed. These new properties are important to construct an accurate analytical model of the hybrid laminates that can then be compared to the measurements of the hybrids.

## 4. Discussion

The chosen hybrid configuration showed the intended ‘catastrophic delamination’ damage-mode, as can be seen in Figure 11. A characteristic load drop at the initial failure of the flax fibres (LS material) was followed by a delamination phase at almost constant stress. The load increase after delamination was not always as pronounced as we hoped. The so-called ‘load reserve’ is the difference between the stress level at delamination $\sigma_{del}$ and the ultimate stress $\sigma_{@HF}$. These values are listed in Table 4.

The detailed analysis of the specimens shown in Figure 13, Figure 14 and Figure 15 show the delamination propagation from an initial failure of the flax layer. This failure can occur anywhere along the specimen, leading to different paths of delamination propagation. In Figure 13b, the delamination starts on the right side, and a second delamination front starts on the left. In Figure 14b, the initial failure of the flax layer occurs on the right side of the plot and then propagates to the left. In Figure 15b, the failure of the flax starts the delamination process from the centre of the specimen. The delamination then propagates outwards in either direction.

From the tensile tests, the load reserve after delamination was calculated to be, on average, 145.5 MPa with a standard deviation of 48.5 MPa. This is the main part that should be improved in further developments. It is important that there be a significant load reserve after delamination. The early warning of the LS material failure requires proper laminate pseudo-ductility, such that the HS material can carry significantly higher loads.

### 4.1. Comparison Between Data and Analytical Model

With the newly determined fibre properties from Table 6, an analytical model designed after Jalalvand et al. [5] can be constructed and compared to the data from Figure 11.

**Figure 17:**
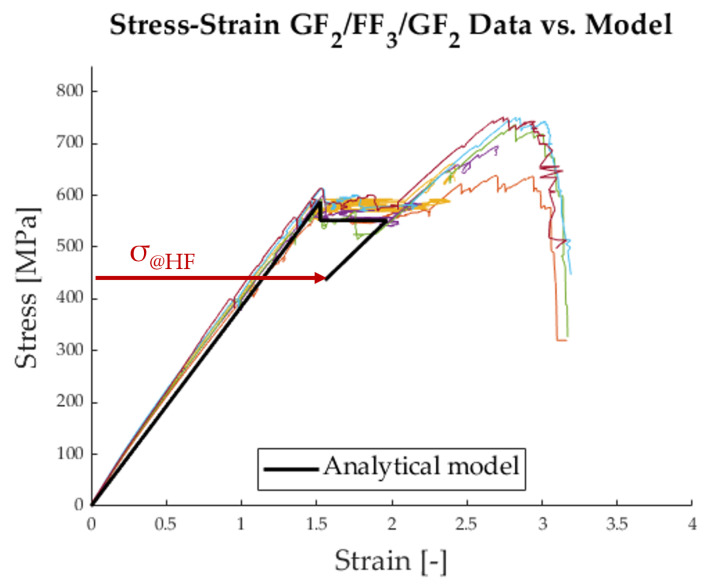
Average strain along the specimen measured with DFOS vs. analytical model [5].

Figure 17 shows that the initial behaviour of the hybrid follows the model well. It is important to note that the stress level $\sigma_{del}$ was fitted to the curve because it is highly dependent on the critical mode 2 energy release rate, which has not yet been determined accurately for this material combination. The chief difference between the model and the data lies in the final failure of the hybrid at the stress level $\sigma_{@HF}$. The analytical model drastically underestimates the strength of the glass fibres. This is due to a reduction term that is applied to the model, following a Weibull strength distribution of the HS material. It can be seen in Equation (6) as $\frac{1}{K_{t}\sqrt[{m}]{V}}$ and is meant to account for the strength distribution within the volume of the HS material [25,26]. For the case at hand, this term is unnecessary because the volume of the specimen, with which the tensile strength was determined, is the same as the volume of the material in the hybrid. Therefore, the model must be adapted as follows:

(8) $$\sigma_{@HF}=\frac{S_{H}}{1+\beta }$$

**Figure 18:**
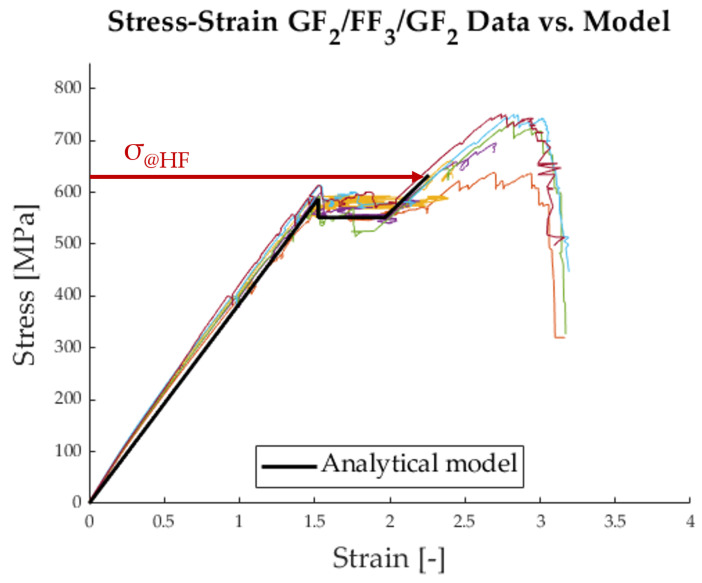
Average strain along the specimen measured with DFOS vs. adjusted analytical model without Weibull strength distribution.

With this adjustment of the model, it is possible to create a new analytical stress–strain curve, which can be seen in Figure 18.

### 4.2. Further Improvements in the Hybrids

**Figure 19:**
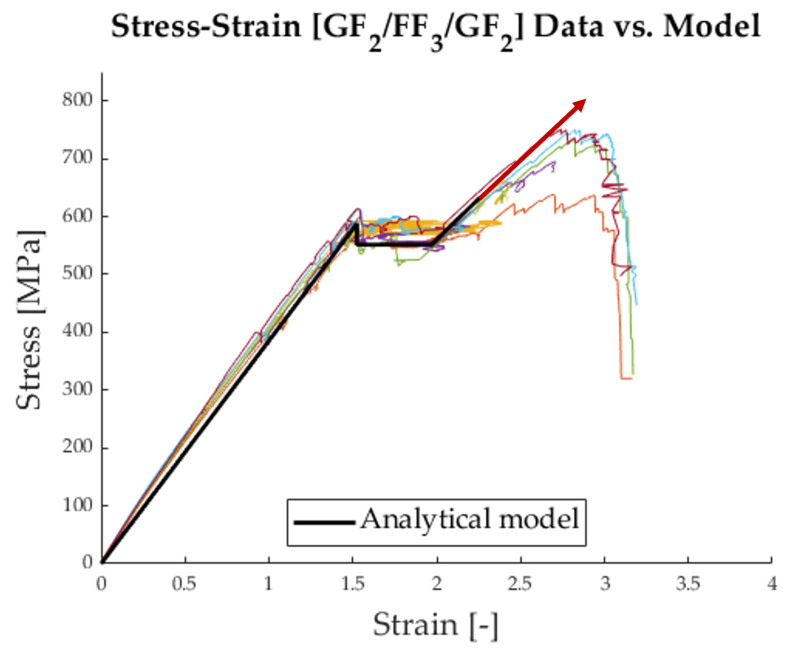
Stress–strain curve, where the red arrow shows the desired, higher load reserve after delamination.

The load reserves of the tested hybrid laminates are neither very high nor consistent. In Figure 13, the load only increases very slightly, whereas in Figure 15, the load increase after delamination is more pronounced. In further investigations, the focus will be on the load reserve and how it can be increased to resemble the stress–strain curve shown in Figure 19.

To increase the load reserve, the tensile strength of the HS material must be increased, this increases the ultimate laminate stress before failure $\sigma_{@HF}$. Different approaches could be pursued to achieve this. By using a different matrix polymer that creates a better interface between fibre and matrix, the strength of the HS material might be improved.

A different approach would be to find a way to remove the weft from the fabric. That way, the stress concentrations due to the waviness of the fibres would be prevented, leading to higher UD-layer moduli and strengths.

Alternatively, the fibre used for the HS material could be changed to achieve higher tensile strength. In this study, an S-Glass UD fabric from China Beihai Fiberglass Co., Ltd. (Jiujiang City, China) was used. There may be other manufacturers that produce S-Glass with higher tensile strength.

## 5. Conclusions

In this study, the potential of pseudo-ductile hybrid composites from flax and glass fibres was investigated. The unidirectional laminates were designed based on an analytical model that could predict the damage-mode and tensile behaviour of a hybrid composite [5]. This model was constructed with the help of preliminary tests of composites that contained only one of the constituents of the hybrid: pure glass/epoxy or pure flax/epoxy. Tensile tests were carried out using both an extensometer and DFOS for the strain measurement. These tests showed the pseudo-ductile behaviour of the designed laminates and thus proved that pseudo-ductility is achievable with this sustainable selection of materials. The specimens showed the desired ’catastrophic delamination’ behaviour with a pseudo-ductile strain of 0.52% [std. dev. 0.09%]. This was followed by an improvable load reserve of 145.5 MPa [std. dev. 48.5 MPa] before final failure of the external glass fibre layers, as was predicted by the model.

The tensile behaviour of flax/glass hybrids could still be further improved by investigating and testing slightly different material parameters, mainly by altering the epoxy, changing to a stronger glass fibre with higher tensile failure strain, using a different fibre product without weft (for the LS and HS materials), investigating the critical mode 2 energy release rate, or changing the layer sequence.

## Figures and Tables

**Table 1:** Points for the construction of the analytical stress–strain model.

Point	Coordinates
1. Origin	(0,0)
2. LS material failure	$\left(\varepsilon_{L},\sigma_{@LF}\right)$
3. Start of delamination	$\left(\varepsilon_{L},\sigma_{del}\right)$
4. Completion of delamination	$\left(\frac{\sigma_{del}}{E_{final}},\sigma_{del}\right)$
5. Final HS material failure	$\left(\frac{\varepsilon_{H}}{K_{t}\sqrt[{m}]{V}},\sigma_{@HF}\right)$

**Table 2:** Dimensions of the laminate.

Dimension	Value
Width, W	24 mm
Total length	280 mm
Free length, L	140 mm
Thickness, t	See Table 3

**Table 3:** Table of all specimens.

Number	Layer Sequence	Thickness	Remark
1	$\left[GF_{2}/FF_{3}/GF_{2}\right]$	2.01	With optical fibres, for tensile test
2	$\left[GF_{2}/FF_{3}/GF_{2}\right]$	1.99	With optical fibres, for tensile test
3	$\left[GF_{2}/FF_{3}/GF_{2}\right]$	2.02	With optical fibres, for tensile test
4	$\left[GF_{2}/FF_{3}/GF_{2}\right]$	2.28	With optical fibres, for tensile test
5	$\left[GF_{2}/FF_{3}/GF_{2}\right]$	1.98	With optical fibres, for tensile test
6	$\left[GF_{2}/FF_{3}/GF_{2}\right]$	1.95	With optical fibres, for tensile test
7	$\left[GF_{2}/FF_{3}/GF_{2}\right]$	2.03	Without optical fibres, for microscopy

**Table 4:** Different stress characteristics of the hybrids from the tests.

Parameter	Value	Std. Dev.
Failure strain of the LS material, $\varepsilon_{L}$ [%]	1.54	0.03
Failure strain of the HS material, $\varepsilon_{H}$ [%]	2.76	0.06
Laminate stress at LS material failure, $\sigma_{@LF}$ [MPa]	599.7	10.6
Laminate stress during delamination, $\sigma_{del}$ [MPa]	567.8	24.1
Laminate stress at HS material failure, $\sigma_{@HF}$ [MPa]	713.9	43.4
Load reserve after delamination [MPa]	145.5	48.5
Pseudo-ductile strain [%]	0.52	0.09

**Table 5:** Summary of measurements of the layer thickness of glass and flax plies, measured from sample 7 under a microscope.

Object	Thickness [mm]	Standard Deviation [mm]	Nr. of Measuring Points
Flax layer (3 plies)	0.97	0.086	82
Glass layer (2 plies)	0.50	0.044	88
Total laminate, t	2.03	0.010	35
**Object**	Derived single-ply thickness *		
Flax ply	0.32		
Glass ply	0.25		

* Calculated from average layer thickness.

**Table 6:** Newly determined laminate properties for glass/epoxy and flax/epoxy.

Property	Flax/Epoxy	Std. Dev.	Glass/Epoxy	Std. Dev.
Young’s modulus, $E_{L,H}$ [GPa]	21.4	3.0	54.9	1.9
Tensile strength, $S_{L,H}$ [MPa]	327.2	32.1	1241.1	57.8
Breaking strain, $\varepsilon_{L,H}$ [-]	1.54%	0.039%	2.95%	0.124%
Fibre volume fraction, $\varphi_{F}$ *	60%	2.3%	66%	1.2%

* The fibre volume fraction was approximated from the thickness of the laminate as follows: $\varphi_{F}=\frac{\rho_{A}/\rho_{V}}{t}$. See Table A3 for densities.

**Figure A1:**
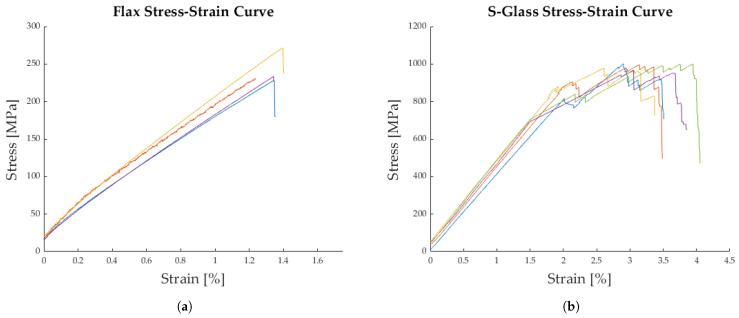
Results of the preliminary tensile tests: (**a**) UD Flax. (**b**) UD S-Glass.

## Data Availability

Data will be made available on request. The datasets generated and analysed during the current study are not publicly available due to institutional policies but may be provided by the corresponding author upon reasonable request.

## Supplementary Materials

The following supporting information can be downloaded at: https://www.mdpi.com/article/10.3390/polym17141953/s1, Video S1: Delamination propagation during tensile test.

## Abbreviations

The following abbreviations are used in this manuscript:

DFOS	Distributed fibre optic sensing
$E_{H}$	Young’s modulus of the HS material
$E_{L}$	Young’s modulus of the LS material
FF	Flax fibre
FRP	Fibre-reinforced polymer
GF	Glass fibre
$G_{IIc}$	Critical mode 2 energy release rate
HS	High-strain
$K_{t}$	Stress concentration factor
L	Length of the specimen
LS	Low-strain
m	Weibull modulus
$S_{H}$	Tensile strength of the HS material
$S_{L}$	Tensile strength of the LS material
$t_{H}$	Half thickness of the HS material
$t_{L}$	Half thickness of the LS material
UD	Unidirectional
W	Width of the specimen
α	$E_{L}/E_{H}$, ratio of Young’s moduli between the LS and HS materials
β	$t_{L}/t_{H}$, ratio of thicknesses between the LS and HS materials
γ	$\frac{t_{L}}{t_{L}+t_{H}}$, LS material fraction
$\varepsilon_{L}$	Breaking strain of the LS material
$\varphi_{F}$	Fibre volume fraction
$\varepsilon_{H}$	Breaking strain of the HS material
$\sigma_{del}$	Laminate stress during delamination
$\sigma_{@HF}$	Laminate stress at HS material failure
$\sigma_{@LF}$	Laminate stress at LS material failure
$\rho_{A}$	Areal density (of the fabric)
$\rho_{V}$	Volumetric density (of the fibres)

## Appendix A

Table A1 and Table A2 summarise the mechanical properties of the unidirectional flax and glass laminates. The corresponding stress–strain curves can be seen in Figure A1. The strain curve of the S-Glass shows an apparent change in stiffness at around 1.5% strain. At this point, the measurement changed from the extensometer (Zwick multiXtens) to machine displacement. The extensometer was detached to avoid damage due to the sudden failure of the GFRP specimens. The stiffness of the specimens appears to be lower after detachment of extensometer, because the machine displacement was influenced by the compliance of the machine. Hence, the machine displacement does not capture the actual specimen deformation alone but also includes the deformation of the machine itself. The flax specimens show another stiffness change at 0.1% strain. This non-linear behaviour is caused by the molecular structure of the flax and was also observed in [18]. Due to this non-linear effect, the Young’s modulus of the flax specimens was averaged from 0% to 1% strain. This differs from the EN ISO 527-5 standard, which was used for all tensile tests in this study.

Table A3 lists the areal and volumetric densities of the dry flax and glass fabrics. These values were used to calculate the fibre volume fractions in Table A1.

**Table A1:** Preliminary, experimental laminate properties.

Property	Flax/Epoxy	Std. Dev.	Glass/Epoxy	Std. Dev.
Young’s modulus, $E_{L,H}$ [GPa]	19.2	2.0	60.3	2.4
Tensile strength, $S_{L,H}$ [MPa]	242.4	20.4	1500.4	15.3
Breaking strain, $\varepsilon_{L,H}$ [-]	1.25%	0.068%	3.75%	0.5%
Layer thickness, $t_{L,H}$ [mm]	0.353	0.030	0.243	0.008
Fibre volume fraction, $\varphi_{F}$ *	60%	4.1%	63%	2.0%

* The fibre volume fraction was approximated from the thickness of the laminate as follows: $\varphi_{F}=\frac{\rho_{A}/\rho_{V}}{t}$. See Table A3 for densities.

**Table A2:** Properties of the laminate.

Property	Laminate
Stress concentration factor, $K_{t}$ [-]	1.08 [3]
Weibull modulus, m [-]	29.3 [3]
Critical energy release rate, $G_{IIc}$ [N/mm]	1.0 [3,27,28]
Length of the specimen, L [mm]	280
Width of the specimen, W [mm]	24

**Table A3:** Areal and volumetric density of the fabrics.

Property	UD Flax Fabric	UD Glass Fabric
Areal density, $\rho_{A}$ [g/m^2^]	300 [21]	414 [20]
Volumetric density, $\rho_{V}$ [kg/m^3^]	1.47 [21]	2.46 [20]
